# Estimating the herd immunity threshold by accounting for the hidden asymptomatics using a COVID-19 specific model

**DOI:** 10.1371/journal.pone.0242132

**Published:** 2020-12-16

**Authors:** Shaurya Kaushal, Abhineet Singh Rajput, Soumyadeep Bhattacharya, M. Vidyasagar, Aloke Kumar, Meher K. Prakash, Santosh Ansumali

**Affiliations:** 1 Jawaharlal Nehru Centre for Advanced Scientific Research, Jakkur, Bangalore, India; 2 Indian Institute of Science, Bengaluru, India; 3 Sankhya Sutra Labs, Manyata Embassy Business Park, Bengaluru, Karnataka, India; 4 Indian Institute of Technology Hyderabad, Kandi, India; 5 VNIR Biotechnologies Pvt Ltd, Bangalore Bioinnovation Center, Helix Biotech Park, Electronic City Phase I, Bangalore, India; Escuela Superior Politecnica del Litoral, ECUADOR

## Abstract

A quantitative COVID-19 model that incorporates hidden asymptomatic patients is developed, and an analytic solution in parametric form is given. The model incorporates the impact of lock-down and resulting spatial migration of population due to announcement of lock-down. A method is presented for estimating the model parameters from real-world data, and it is shown that the various phases in the observed epidemiological data are captured well. It is shown that increase of infections slows down and herd immunity is achieved when active symptomatic patients are 10-25% of the population for the four countries we studied. Finally, a method for estimating the number of asymptomatic patients, who have been the key hidden link in the spread of the infections, is presented.

## Introduction

COVID-19 infections have breached the five million mark, yet there is neither a vaccine nor a scalable treatment in sight [1, 2]. Furthermore, a distinctive feature of the COVID-19, in contrast to other infectious diseases such as Influenza or SARS, is the presence of a large fraction of “asymptomatic” patients, who don’t have any obvious symptoms but are still capable of infecting susceptible individuals through contacts. However, identifying individuals spreading infections via the asymptomatic pathway is not easy unless extensive contact tracing and testing is performed. A major challenge is the uncertainty in the estimation of asymptomatic fraction, with estimates ranging from 41% to 86% of infected [3, 4]. And along the symptomatic pathway, 44% [5] to 68% [6] of the infections are spread before the onset of symptoms rendering the quarantining people with symptoms less efficient compared to other infectious diseases. These challenges have driven governments to implement non-pharmaceutical interventions (NPIs) such as social distancing and partial or full lock-downs [7]. An unsaid, *a posteriori*, rationale for these lock-downs is that they provide efficient isolation mechanism for the asymptomatic. However, a dearth of quantitative understanding of the effects of the lock-down has triggered debate around the effectiveness, duration and mode (partial vs. full) of lock-down. Thus, it is even suggested that societies should just move in an unhindered manner, towards the attainment of the “herd-immunity threshold” [8]. This threshold is achieved when a sufficiently large proportion of a population becomes immune, and as a result, the disease spread slows down. For COVID-19, estimating the onset of herd immunity remains elusive, and indeed, ascertaining whether herd immunity exists at all! Moreover, high case fatality rate of 3–10% (vs. 0.05% for seasonal influenza) limits the practicality of herd immunity as an effective policy tool. Thus, models that can provide quantitative estimates of the disease spread and the impact of policy measures are expeditiously required.

Similar to other epidemics/pandemic, three different kinds of models are used for COVID-19: 1) Statistical extrapolation models which fit the observed patterns of infections to make short-term prediction [9, 10], 2) Agent based models for a qualitative illustration of microscopic dynamics of spreading infections [11], and 3) Compartment models which divide the population into groups based on the current different disease state of the individual and model the interaction among them [2, 12–14]. Since 1927 plague in Mumbai, compartmental models have been a standard guiding tool for policy decisions [15]. The spread of flu-like diseases (influenza, SARS, COVID-19 etc) is often modelled using three or four compartments: Susceptible-Infected-Recovered (*SIR*) or Susceptible-Exposed-Infected-Recovered (SEIR). Some variants, also consider theoretically a simple containment option, of quarantining infected persons with symptoms. However, all these models assume that only contact between the *S* and the *I* compartments leads to new infections, with the implicit assumption that contact between the *S* and *E* compartments does not lead to any infection. In contrast, an asymptomatic patient with COVID-19 can, and does, infect susceptible individuals through contact. Thus, epidemiological models must consider the distinction between asymptomatic and symptomatic. Moreover, models should distinguish between lock-down and quarantine as these are two qualitatively different policy tools the former operating at the level of a society and the latter the level of a few individuals.

In this paper, we aim to model all these novel aspects of COVID-19 and accomplish three goals:

1. Formulate a minimal epidemiological model incorporating the above mentioned unique aspects of COVID-19 disease spread and associated policies. We accomplish this by formulating a *SAIR* model by including the asymptomatics (*A*) and suitably adapting the governing equations. Since lock-down was unique to COVID-19 management, we also include it in an explicit fashion by using discontinuous in time reproduction rate (the effective rate at which susceptible population get converted into infected).
2. Establish that the model representatively captures the observed epidemiological data, and sheds light on the underlying parameters and universalities that govern the dynamics in the different phases of the pandemic spread and containment. We accomplish this by deriving a closed-form solution for the *SAIR* model with and without lock-downs, and using the solution to estimate the underlying parameters that drive the infection dynamics.
3. Use the model to address pertinent questions beyond what is readily measurable—estimates of the hidden asymptomatics or at what fraction of symptomatic infections, herd immunity would be achieved. We show that the for the countries we studied, the herd immunity for COVID-19 is achieved when the symptomatic infections is around 10–25% of the population, which is lower than estimated/expected/what was traditionally assumed.

## COVID-19 specific model

### Unique character of infectiousness

**Fig 1:**
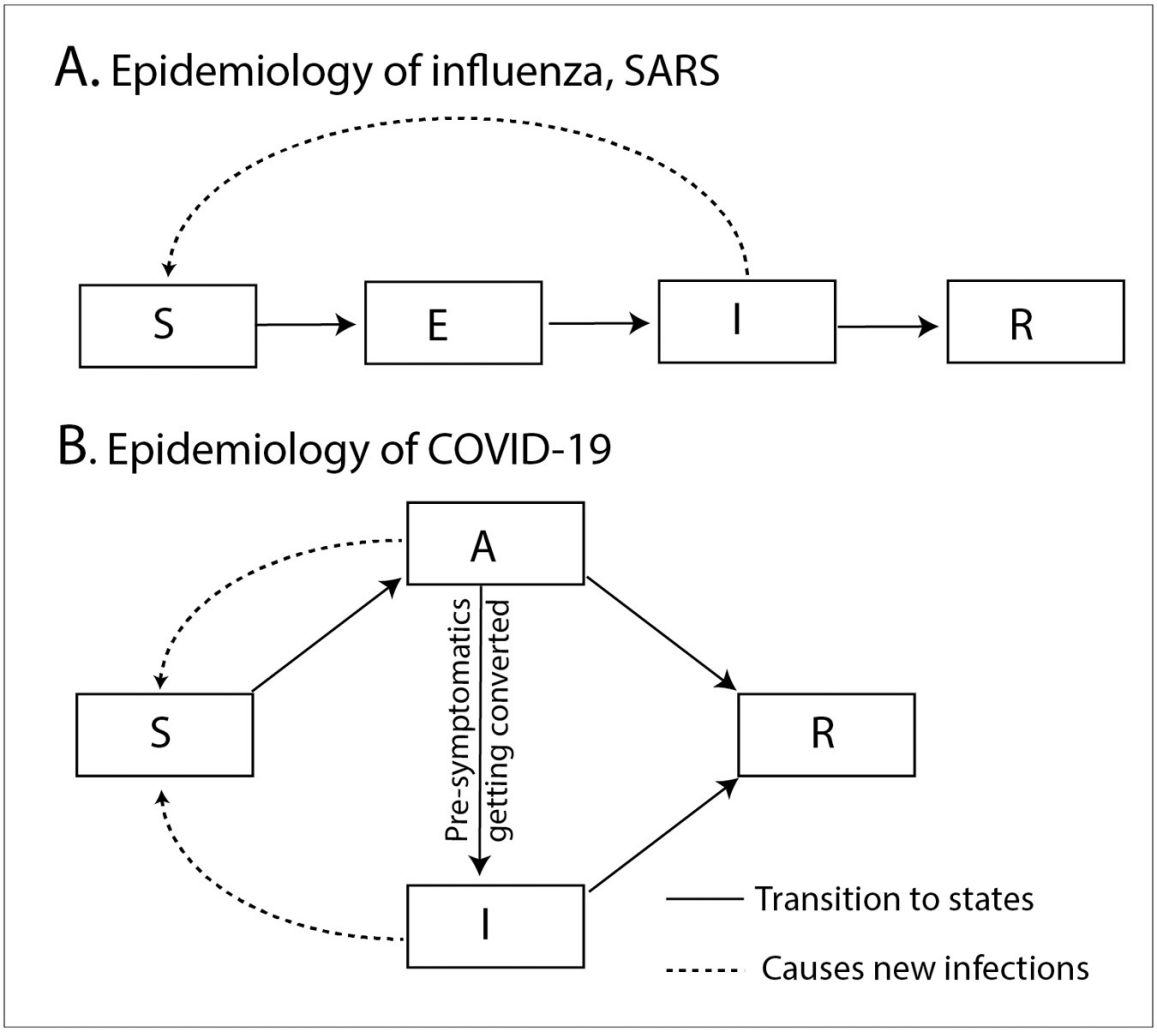
Schematic illustrating the difference between the standard *SEIR* model and the *SAIR* model used in this work. While the former is a convenient model that is commonly used for other respiratory infections, the latter captures the epidemiological characteristics of the COVID-19 infection spread.

We begin by emphasizing the difference between *SEIR* and *SAIR* models [12]. A typical *SEIR* model assumes a framework of serial, directed transitions across the intermediate health states of the individuals (Fig 1). In this framework, the infections are caused when a susceptible person comes in contact with a person deemed to be infected person on the basis of the symptoms (I). However, after this contact, with a certain likelihood the person remains in a pre-symptomatic intermediate state or the exposed individual (*E*), that is not contagious, before transitioning to a contagious and symptomic state (*I*). While this framework is acceptable for influenza or SARS, the epidemiology of COVID-19 is such that there is an alternative pathway between the susceptible (*S*) and the recovered states (*R*) which passes through asymptomatic individuals (estimated to be around 86%), [3] who never show any symptoms but carry enough viral load to infect others. Thus a model for COVID-19 should consider two parallel pathways of infection (Fig 1B).

We develop a generalized version of the model considering the Susceptible, Asymptomatic, Infected and Recovered compartments (*SAIR* model) and assuming a homogenously mixed population segment where COVID-19 is spreading. The system will obey the following *SAIR* dynamics

$$\begin{array}{c} \begin{array}{cc} \dot{S} & =-\alpha \left(t\right)\;S(I+A),\\ \dot{A} & =\alpha \left(t\right)\;S(I+A)-\delta \;A-\gamma A+\beta \left(t\right)A,\\ \dot{I} & =\delta \;A-\gamma I,\\ \dot{R} & =\gamma \;(I+A). \end{array} \end{array}$$ (1)

where for any variable *X* time derivative is denoted as $\dot{X}=dX/dt$. We assume that *α*(*t*) denotes the probability with which, when a susceptible person meets an infected or asymptomatic person, they become a part of the asymptomatics, which for simplicity includes the pre-symptomatics and the asymptomatics. *δ* is the rate at which pre-symptomatics or asymptomatic patients get converted into symptomatic ones and *γ* is the rate at which both asymptomatic and symptomatic patients recover. *β*(*t*) is a migration parameter, the origin and exact functional form of which will be discussed in the next section.

### Lock-down for infection containment

In our formulation of the model, we claim that the lock-down can be modeled by considering a sudden change in the infection rate constant using a Heavyside function as $\alpha \left(t\right)=\alpha_{0}H\left(t_{\text{lock}}-t\right)$. Here, we note in passing that one can model social distancing as reduction in value of *α* or an imperfect lock-down. This term, typically absent in standard models, denotes the fact that in an idealized lock-down no susceptible person meets an infected person and thus first order reaction changes to a zero-order reaction. In a minimal model, one may assume that asymptomatic patients either get converted into symptomatic one with an effective rate *δ* or recovers with a rate *γ*.

Before we proceed to analyse the model, we wish to point out that one may add further complication to this model by introducing more parameters and compartments. For example, recovery rate *γ* and infection rate *α* need not be same for asymptomatic and symptomatic fraction [12]. However, as there is no biological evidence to the contrary, we assume that both rates are equal, which leads to an analytically tractable and simplified framework.

Further, during this crisis we learnt that once a lock-down is announced, people migrate across different segments of a country. Even for a qualitatively correct modeling of disease spread dynamics, it is important to account for this migration of people. This migration can indeed happen in many waves. However, for simplicity we assume that it happens once and only during a short duration after lock-down. Furthermore, one would expect that among infected population only asymptomatic people are able to travel. Here, it needs to be reminded that, we are only interested in the influx of the infected population in a given population segment, and not the details of where they came from. In order to model such a scenario, we take typical thermodynamic route of dividing the system into two parts: system (Eq (1)) and universe (given in Eq (2)). Finally, the coupling constant $\beta \left(t\right)=\beta_{1}\{H\left(t_{\text{lock}}+\epsilon -t\right)-H\left(t_{\text{lock}}-t\right)\}$ and *ϵ* is the short period of time post lock-down, in which population migration is allowed/possible. For the sake conceptual completeness of showing where these migrations happen from, one can also write the equations for the net of all geographical regions outside the region of interest (which for the sake of convenience is described as the rest of the Universe (U)). This migration is a characteristic of the system (country or region under consideration) and parameters *β* and *ϵ* need to be extracted from the data. The universe (rest of the world) can also be assumed for this purpose to be following a similar *SAIR* dynamics, but with an outflux term (−*β*(*t*)*A*) in the asymptomatic dynamics

$$\begin{array}{c} \begin{array}{cc} {\dot{S}}_{\text{U}} & =-\alpha \left(t\right)S_{\text{U}}\;(I_{\text{U}}+A_{\text{U}}),\\ {\dot{A}}_{\text{U}} & =\alpha \left(t\right)S_{\text{U}}\;(I_{\text{U}}+A_{\text{U}})-\delta \;A_{\text{U}}-\gamma A_{\text{U}}-\beta \left(t\right)A,\\ {\dot{I}}_{\text{U}} & =\delta A_{\text{U}}-\gamma I_{\text{U}},\\ {\dot{R}}_{\text{U}} & =\gamma \;(I_{\text{U}}+A_{\text{U}}). \end{array} \end{array}$$ (2)

Eqs (1) and (2) complete our development of COVID-19 specific model. However, in the rest of this work, we will focus on solving Eq (1).

### Analytical solution of COVID-19 model without lock-downs

In the present work, we solved a phenomenological model of a well-mixed society, with everyone interacting with everyone else. However, the interactions may be structured by age, local movement of the population, and many of these can be modelled in the framework of agent based models. The formulation of the disease specific interactions we developed can also be integrated into other models which study the interactions at agent level detail, or in tandem with economic consequences [16], both of which are beyond the scope of the present work. With an emphasis mainly on the spread of infections at the societal level, we show that the set of equations we model are sufficient to capture most of the available epidemiological data on COVID-19.

This system of equations can be solved for pre-lock-down situation in terms of the reproduction rate *r*_0_ = *α*_0_/*γ* by defining *M* = *I* + *A*.

$$\begin{array}{c} \frac{d\;\text{log}\;S}{dR}=-r_{0},\;\frac{dM}{dS}=-1+\frac{1}{Sr_{0}}. \end{array}$$ (3)

which can be solved in terms of $\tilde{S}=S/S_{0}$ as

$$\begin{array}{c} R=-r_{0}^{-1}\;\text{log}\;\tilde{S},\;\;M=1-S+r_{0}^{-1}\;\text{log}\;\tilde{S} \end{array}$$ (4)

where *M* + *S* + *R* = 1 when there is no influx of people into a country, *S*_0_ denotes the susceptible population at *t* = 0 and the recovered population at *t* = 0 is taken to be 0. Substituting the expression from Eq (4) in the evolution equation for *S* gives us the parametric solution in implicit form as

$$\begin{array}{c} \alpha_{0}\;t=\int_{1}^{\tilde{S}}\frac{ds}{s\;(-1+S_{0}s-r_{0}^{-1}\;\text{log}\;s)} \end{array}$$ (5)

Assuming that the equation can be converted to an explicit form for $\tilde{S}$ as a function of *t*, it is possible to substitute this into Eq (4) to obtain an expression for *M* as a function of *t*. Finally, the expression for *M*(*t*) can be disambiguated into separate expressions for *I*(*t*) and *A*(*t*) by using Eq (1). Specifically, in the equation for $\dot{I}$, we can substitute *A* = *M* − *I*, which gives

$$\begin{array}{c} \dot{I}=-(\delta +\gamma )I\left(t\right)+\delta M\left(t\right). \end{array}$$

If we define a new constant *δ*_1_ = *γ* + *δ*, then the solution of the above equation is

$$\begin{array}{c} I\left(t\right)=\text{exp}\left(-\delta_{1}t\right)[I_{0}+\delta \int_{0}^{t}M\left(s\right)\;\text{exp}\left(\delta_{1}s\right)ds] \end{array}$$ (6)

**Fig 2:**
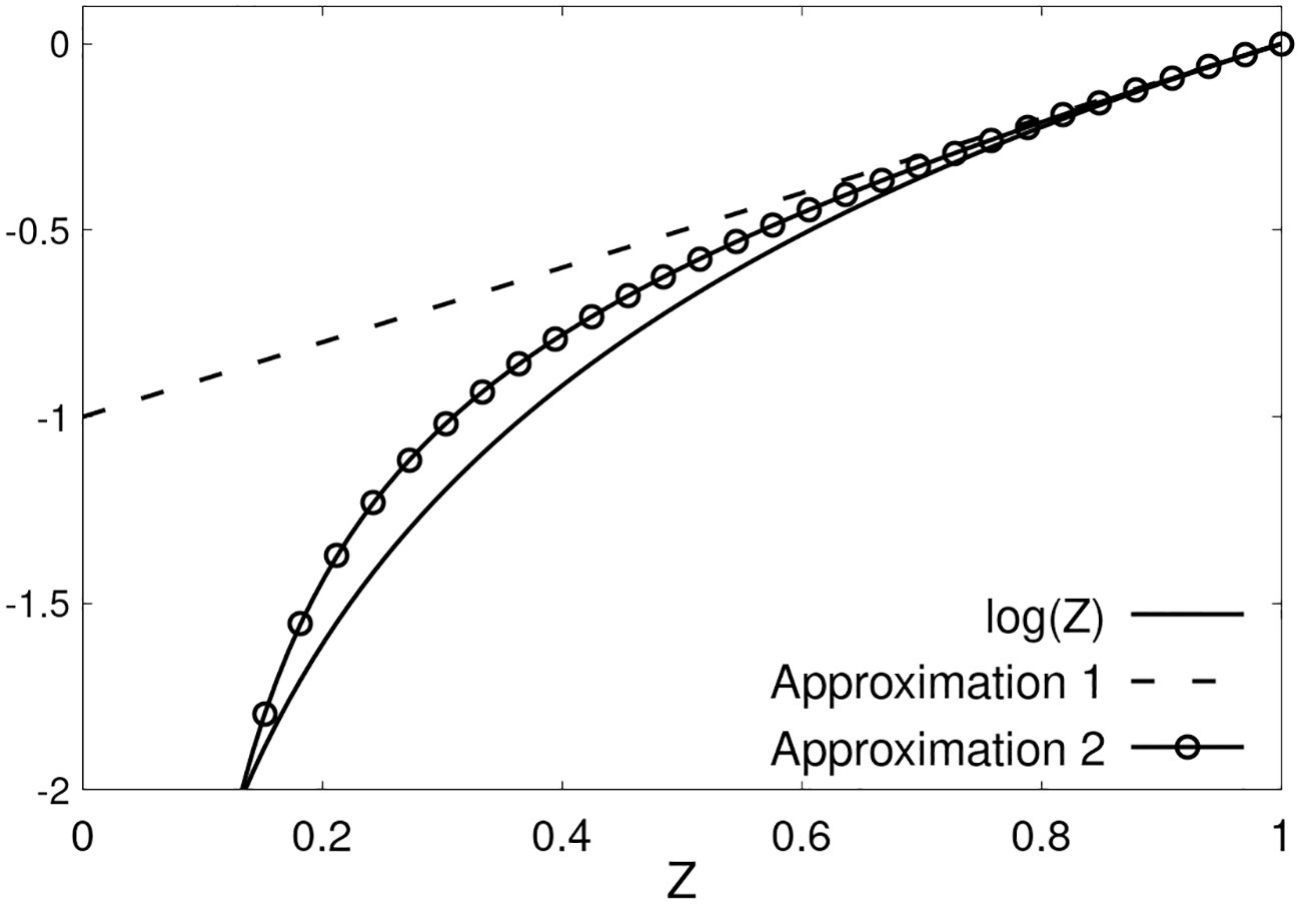
Comparison between the two approximations for log(*z*). Approximation 1 log(*z*) ≈ (*z* − 1) and approximation 2 is log(*z*) ≈ (*z* − 1)(*w*_1_/*z* + *w*_2_), where *w*_1_ = 1/5 and *w*_2_ = 4/5.

**Fig 3:**
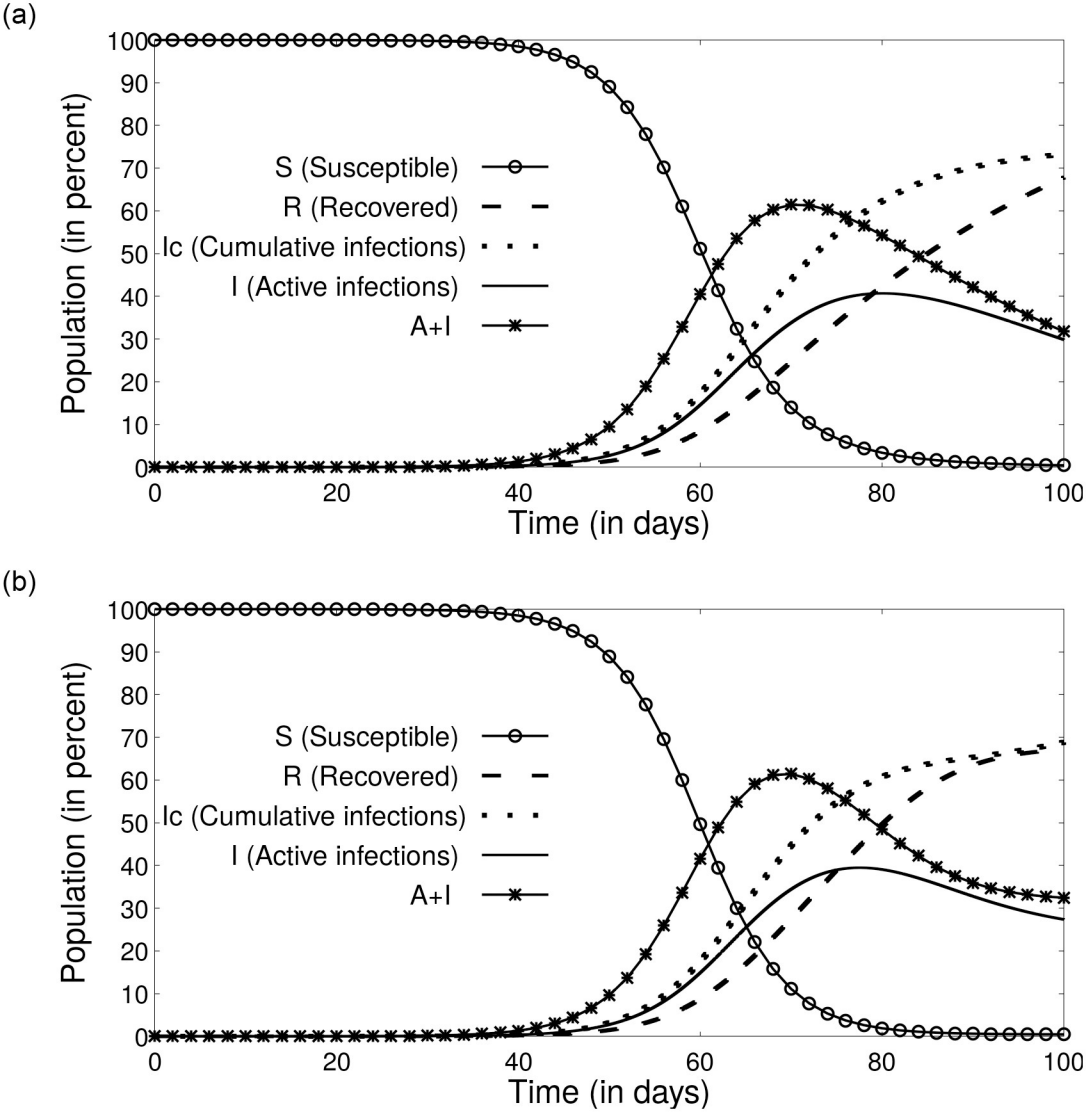
Solutions to the SAIR model without lock-down: (a) Numerical solution to the ODE. (b) Analytical solution to the ODE using approximation 2. The parameters used in this simulation are the ones we obtained from the epidemiological data of Italy (Table I): *α*_0_ = 0.256, *γ* = 0.03 and *δ* = 0.09. As one can see, the numerical and analytical solutions yield comparable results.

Therefore the key is to turn Eq (5) into an explicit expression, to the extent possible. For this purpose, we use Hermite-Hadamard inequality for the logarithm [17]

$$\begin{array}{c} \frac{z-1}{z}\leq \text{log}\;z\leq z-1 \end{array}$$ (7)

which suggests that we use approximate form of the logarithm as log *z* = (*z* − 1)(*w*_1_/*z* + *w*_2_), with the constraint that *w*_1_ + *w*_2_ = 1. Fig 2 depicts the ability of the approximatrion to capture log(*z*) when 0 < *z* < 1. Upon approximating the logarithm, we get a solution in explicit form as

$$\begin{array}{c} \tilde{S}=\frac{h(1+h_{2}\;\text{exp}\left(h\;\alpha_{0}t\right))}{2a(1-h_{2}\;\text{exp}\left(h\;\alpha_{0}t\right))}+\frac{b}{2a} \end{array}$$ (8)

where *a* = (*S*_0_ *r*_0_ − *w*_2_)/*r*_0_, *b* = (*r*_0_ + *w*_1_ − *w*_2_)/*r*_0_, *d* = *w*_1_/*r*_0_. where *h*_2_ = (2*a* − *b* − *h*)/(2*a* − *b* + *h*), and *h* is a constant such that and $h=\sqrt{b^{2}-4ad}$. Once the evolution equation for *S* is known in a closed from, we find the evolution for the remaining variables using Eq (4). Fig 3a and 3b) depicts a representative temporal variation for the parameters *S*, *A*, *I* and *R* captured using the numerical and analytical solution. The analytical solution formulated using the above approximation to logarithm is found to be in close agreement with the numerical solution of the ODE.

The evolution equations for pre-lock-down and early time limit is easily formulated by incorporating approximation 1 for the logarithm, leaving us with

$$\begin{array}{c} \tilde{S}=\frac{r_{0}-1}{r_{0}\left(1-S_{0}\right)\;\text{exp}\left\{\alpha_{0}\left(1-r_{0}^{-1}\right)t\right\}-\left(1-r_{0}S_{0}\right)} \end{array}$$ (9)

and

$$\begin{array}{c} I=\frac{\left(S_{0}-1\right)\left(r_{0}-1\right)\;\;\text{exp}\left\{\left(\alpha_{0}-\gamma \right)t\right\}}{2-2r_{0}S_{0}+2r_{0}\left(S_{0}-1\right)\;\;\text{exp}\left\{\left(\alpha_{0}-\gamma \right)t\right\}} \end{array}$$ (10)

### Analytical solution of COVID-19 model with lock-downs

On the other hand, after an idealized lock-down no susceptible person meets an infected person and thus the first order reaction changes to a zero order reaction. The intermediate time (*t*_lock_ < *t* < *t*_lock_ + *ϵ*) solution simplifies to

$$\begin{array}{c} M=\text{exp}\left(-\gamma t\right)[I_{\text{lock}}+A_{\text{lock}}\left(1+\delta \right)\;\text{exp}\left(\left(\beta_{1}-\delta \right)t\right)] \end{array}$$ (11)

Once there is no more flux of asymptomatic individuals (i.e. after *t*_lock_ + *ϵ* days), the equations for *M* yield an exponential decay given by

$$\begin{array}{c} M=M_{\text{lock}+\epsilon }\;\;\text{exp}\left(-\gamma t\right),\;\;R=1-M-S_{\text{lock}} \end{array}$$ (12)

and the infections post lock-down is given by

$$\begin{array}{c} \begin{array}{cc} I & =\text{exp}\left\{-\gamma t\right\}\left(I_{\text{lock}}-L\right)\\ & +L\;\text{exp}\{\left(-\delta_{1}+\beta_{1}\left(1-H\left(t-\epsilon \right)\right)\right)t\} \end{array} \end{array}$$ (13)

where,

$$\begin{array}{c} L=\frac{\delta (A_{\text{lock}}\;\text{exp}\left\{\epsilon \left(\beta_{1}-\delta_{1}\right)H\left(t-\epsilon \right)\right\})}{\left(\gamma -\delta_{1}\right)+\beta_{1}\left(1-H\left(t-\epsilon \right)\right)} \end{array}$$ (14)

The recovery rate is split into two parts

$$\begin{array}{c} \dot{R}=\gamma I+\gamma A={\dot{R}}_{I}+{\dot{R}}_{A} \end{array}$$ (15)

The recovery of infected people is easier to track for any country and hence is the more important equation for parameter extraction.

$$\begin{array}{c} \int_{0}^{t}{\dot{R}}_{I}=\gamma \int_{0}^{t}I \end{array}$$ (16)

Eqs (10), (12), (13) and (16) are the closed-form solutions to the model we developed and are used in the next section for parameter estimation.

### SAIR to SIR

One may think of SIR model as a coarse-grained version of the SAIR model formally. In the coarse grained picture of SIR model, one does not make a distinction between asymptomatic and symptomatic. Thus, it is natural to expect that the total infection $\tilde{I}=A+I$ shall follow the SIR dynamics. Indeed, upon ignoring the migration effects (*β* = 0), we arrive at the SIR model in term of $\tilde{I}$ as

$$\begin{array}{c} \begin{array}{cc} \dot{S} & =-\alpha \left(t\right)S\tilde{I}\\ \dot{\tilde{I}} & =\alpha \left(t\right)S\left(A+I\right)-\gamma \tilde{I}\\ \dot{R} & =\gamma \tilde{I} \end{array} \end{array}$$ (17)

We would like to insist that despite this formal relationship between the model, the two model represent fundamentally two different dynamics in reality. This is largely due to the fact that $\tilde{I}$ is not an observable and one can not get any information on it by looking at reported infection number unless full contact tracing is implemented to test even asymptomatic fraction. Thus, although it may be possible to arrive at an *SIR* model from *SAIR* under certain conditions, the reverse is not the same. Nevertheless, in these limits where this conversion and comparison to *SIR* model is possible, we demonstrate the quality of our approximate solution.

**Fig 4:**
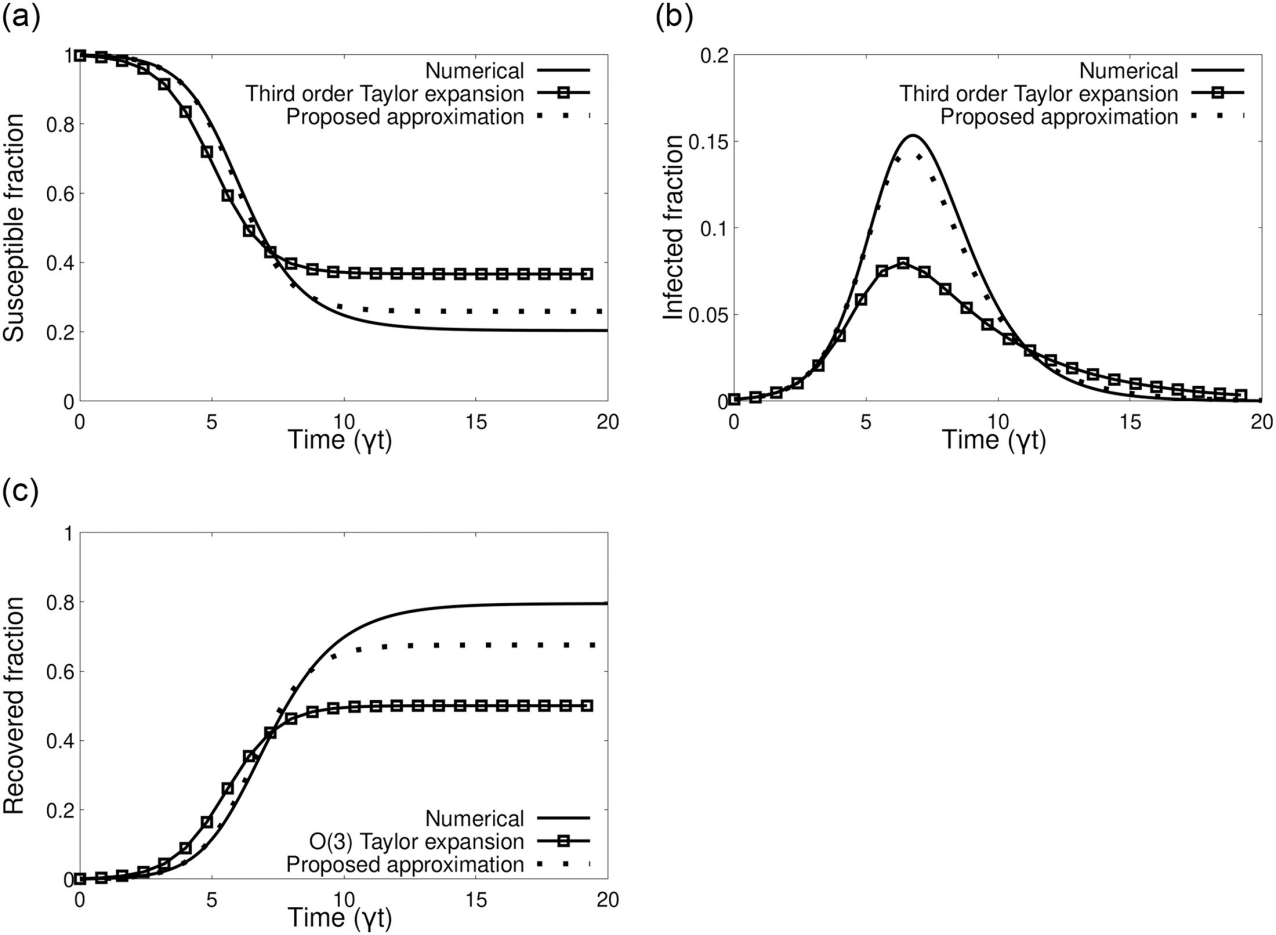
SIR model with *r*_0_ = 2, *I*_0_ = 10^−3^ and *R*_0_ = 0. a) Susceptible fraction b) Infected fraction c) Recovered fraction. The difference of using a third order Taylor series approximation as given in [18] versus our approximation in Eq (5) is shown.

Fig 4 shows that the proposed approximation in Eq (5) yields more accurate results than a third order Taylor series approximation for the same [18]. A more elaborate contrast between present result and various other approximations [18–20] is left for future project.

## Discussion

### Closed form solution

COVID-19 is unique in many ways. In this we work we attempt to capture two major differences as regards its epidemiology—the dynamical aspects of infections spread through pre-symptomatic and asymptomatic persons, and that containments are mainly achieved by lock-downs at the level of the society rather than quarantines. Our model and its closed form-solution address these unique aspects that COVID-19 presented itself with. Epidemics like SARS in 2003, Swine flu in 2009, MERS in 2012 and 2015, could be managed at most with contact tracing and quarantine, and hence addressing a solution for the lock-down did not arise with earlier epidemics. As can be imagined, the closed form solution makes both the prediction and the assumptions/parameters involved in the prediction transparent, which can otherwise be buried in the numerics. In addition, since when we work with real data, rather than use *a priori* assumptions, the formulation helps an easy extraction of the underlying parameters as it is done below.

### Parameters and their universality

**Fig 5:**
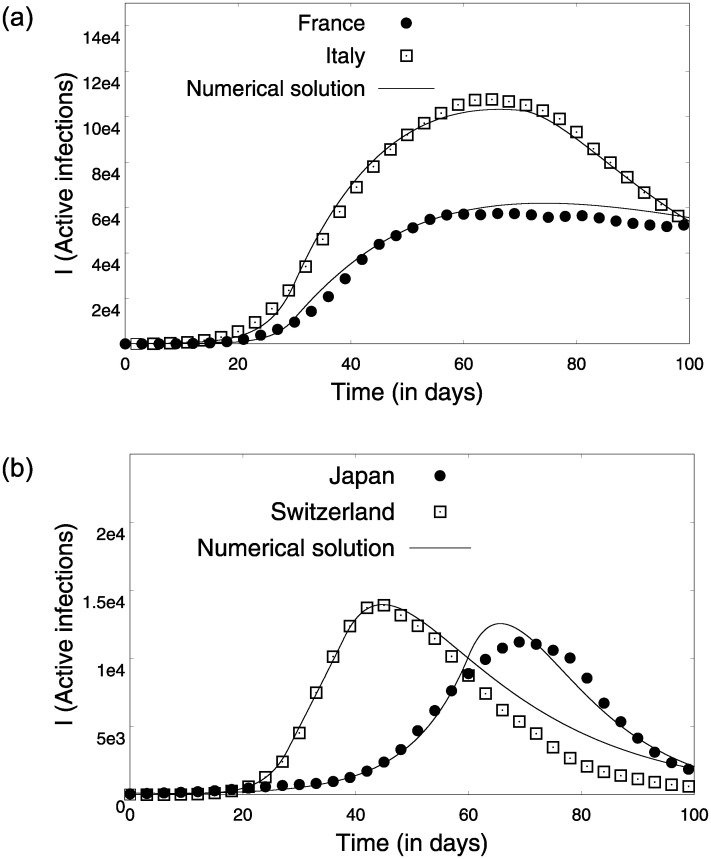
Numerical solution of the SAIR model in solid lines plotted along with the infection data from (a) France and Italy, (b) Japan and Switzerland. The points indicate the 7-day average of the epidemiological data obtained between the dates 14th February 2020 and 24th of May 2020.

**Fig 6:**
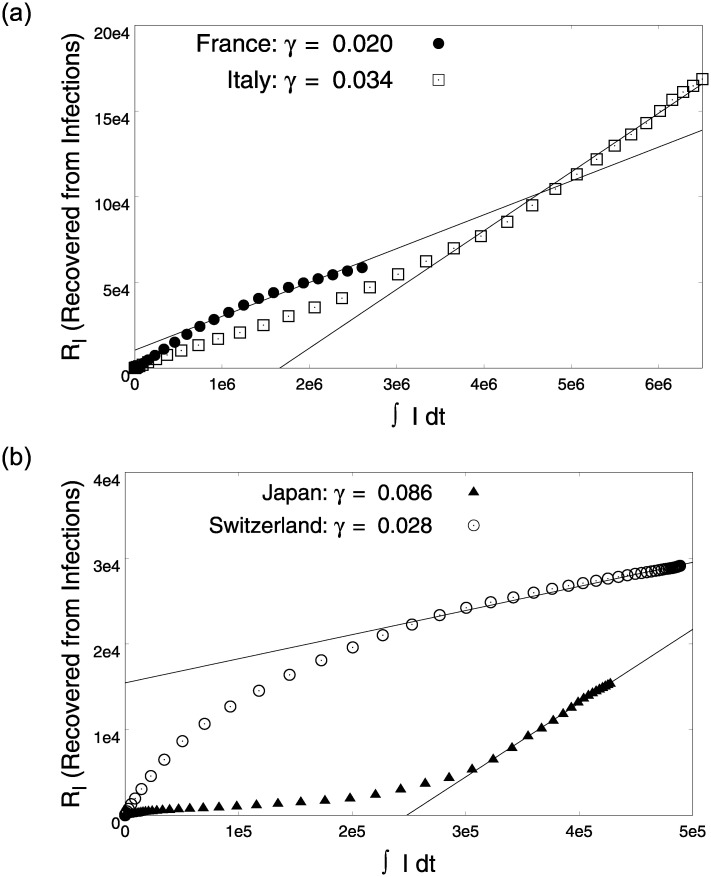
Estimation of parameter *γ* using Eq (16). The linear parts of the data are used for obtaining these fits.

The publicly available reported infection and death data from different countries was gathered from the worldometer site (https://worldometers.info). The infection data from different countries that implemented a lock-down had three regimes—rising, intermediate and decreasing. It can be easily assumed that the reported infections are the symptomatic infections, since most countries have been short of testing resources; as a result, patients were tested for a confirmation only after the onset of symptoms. The analytical solutions for the active infections and recovered populations for both pre- and post- lock-down scenarios discussed above model these different regimes of the infection spread. These analytical solutions are then fit onto the reported infections for several countries Fig 5, to give us an estimate of the underlying parameters that govern the COVID19 infection dynamics. In this section we discuss the estimation procedure for the parameters (*α*_0_, *γ*, *δ*, *β*_1_). We begin with Eq (16) as the data for recovered patients and active infections is readily available and a simple linear fit gives us an estimate for *γ* as shown in Fig 6. *γ*^−1^ is a measure of the average number of days it takes for a COVID-19 infected person to recover. The values of *γ*^−1^ found for France, Italy, Japan and Switzerland lie within a universal sensible range (2-4 weeks) observed for COVID-19.

**Fig 7:**
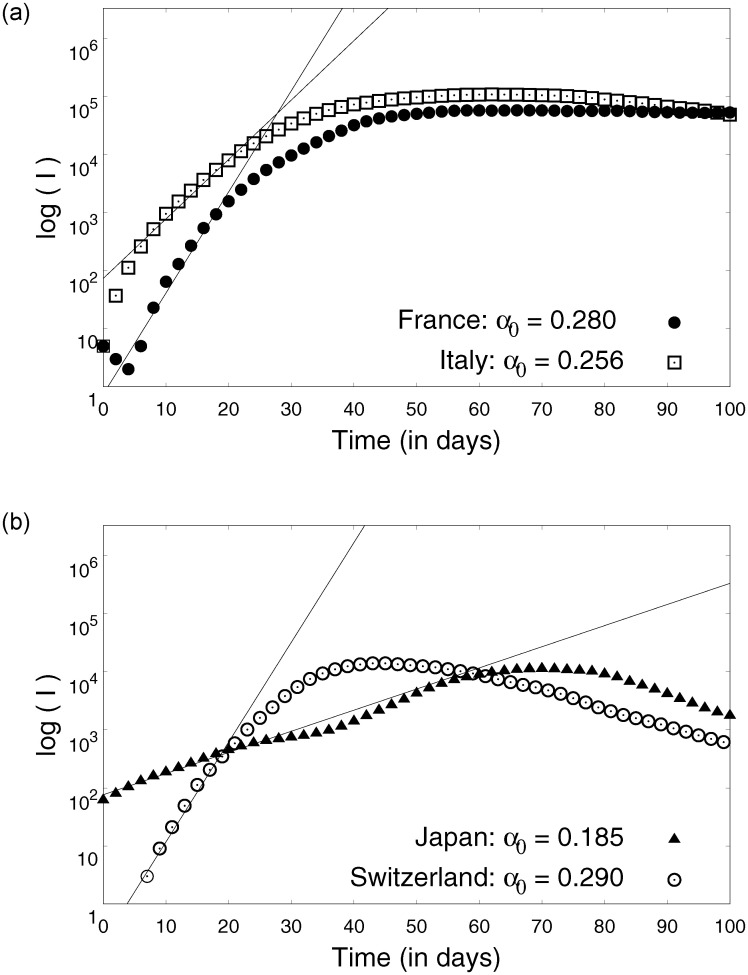
Estimation of parameter *α*_0_ using Eq (10). The linear parts of the data were used for obtaining these fits.

**Fig 8:**
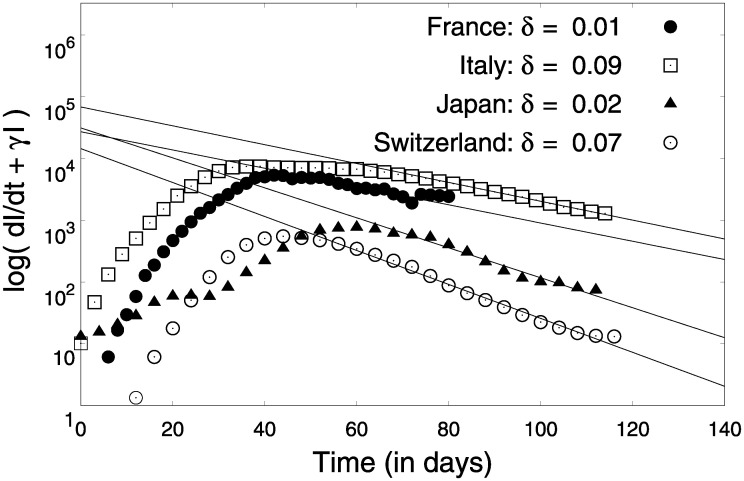
Estimation of parameter *δ* using Eqs (12) and (13). The linear parts of the data were used for obtaining these fits.

**Fig 9:**
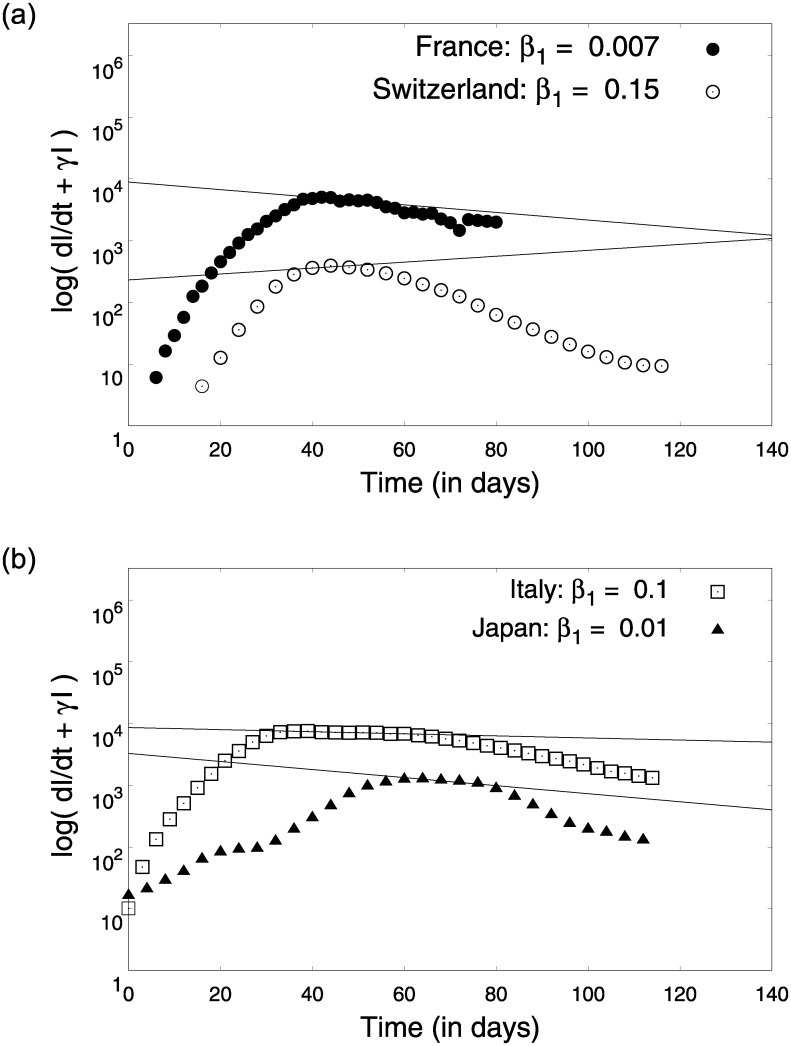
Estimation of parameter *β*_1_ using Eqs (12) and (13). The linear intermediate parts of the data were used to obtain these fits.

Using Eq (10) and real time infection data, parameter *α*_0_ can be estimated as shown in Fig 7. Fig 7 reveals that the three different regimes of the COVID19 infection dynamics could be modelled by the framework we developed. The parameters *α*_0_ representing the rise is similar for many countries reiterating a universal pattern in the initial pre-lock-down regime. This can be understood as an intrinsic characteristic dynamics of COVID-19 which exhibits strong similarities across countries (see Table 1) Using the simplified equations post lock-down (Eqs (11) and (12)), estimated parameter *γ* and $\left(\dot{I}+\gamma I\right)$ data for different countries, we estimate the parameters *δ* and *β*_1_, as shown in Figs 8 and 9 respectively. The origin of *β*_1_ is the migration of people during lock-down and can be expected to be a country-specific event dictated by the prevalent social-political conditions.

**Table 1:** Parameters extracted by fitting the analytical solutions to the model we developed to the 7-day average data from the different countries.

Country	*α*_0_	*γ*	*δ*	*β*_1_
**France**	0.280 ± 0.012	0.021 ± 0.004	0.010 ± 0.001	0.007 ± 0.0005
**Italy**	0.256 ± 0.021	0.034 ± 0.002	0.091 ± 0.007	0.102 ± 0.002
**Japan**	0.185 ± 0.013	0.086 ± 0.003	0.022 ± 0.001	0.012 ± 0.004
**Switzerland**	0.290 ± 0.009	0.028 ± 0.001	0.024 ± 0.002	0.151 ± 0.02

### Asymptomatic infections

**Fig 10:**
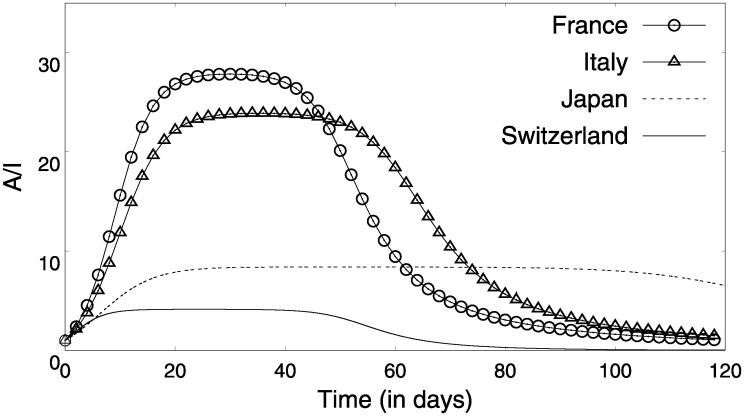
The ratio of asymptomatics to the infected population as a function of time, and for a no-lock-down scenario.

So far we have made predictions based on the COVID19 specific model we developed and validated its efficacy by showing that the analytical solution provides very good fits to the three different regimes of infection. Following up on these validations from the observed infections, we use the model to estimate the number of asymptomatic people, an extremely important metric in quantifying the extent of COVID19 spread. Using our model and the parameters that were extracted, we could estimate how the number of individuals in the different compartments *S*, *A*, *I* and *R* changed with time with or without a lock-down. We estimate the ratio of the asymptomatic to symptomatic individuals (Fig 10), which varies from 1 to 30 depending on the phase of the pandemic dynamics. Seroprevalence tests on small population samples of a few hundreds to a few thousands have estimated the asymptomatics to be present in excess of symptomatics by a factor of 5.7 in Geneva, Switzerland [21], 20 in Wuhan, China [22] to about 50 in Santa Clara, USA [23]. In practice, it has not yet been possible to perform tests on more than a few thousands from cities with millions in population. In that sense, the reliability of the serological tests can be established based on the randomness, and representativeness of the community. However, the predictions of the asymptomatics to symptomatics extracted using our model is in a comparable range, thus serving as a mutual validation, for our predictions and the small serological sample sets.

### Herd immunity

**Fig 11:**
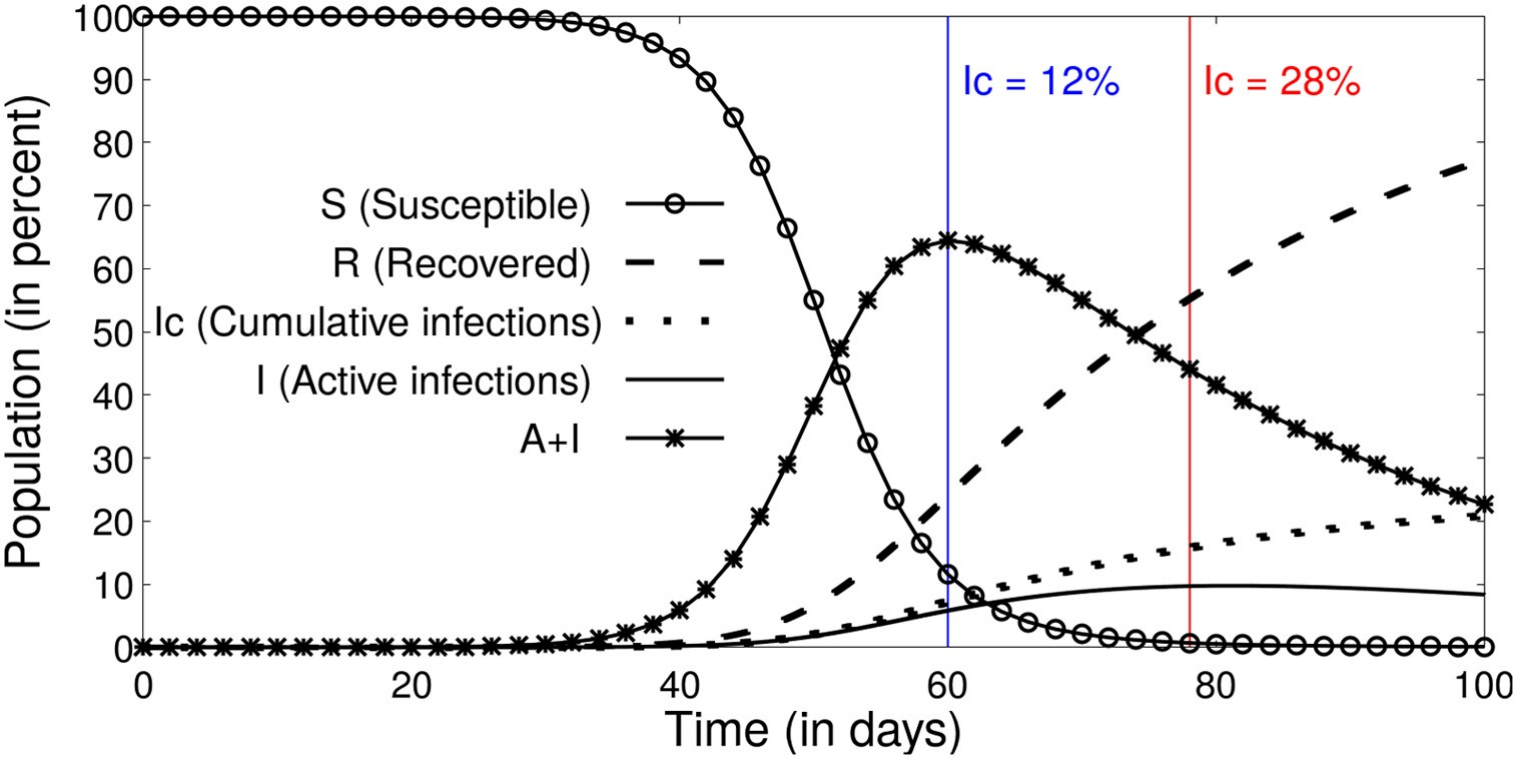
Analytical solution of the SAIR model with no lock-down using parameters for France (*α*_0_ = 0.28, *γ* = 0.02, *δ* = 0.01. The blue and the red lines indicate the maxima, considering only the symptomatic or the total infections respectively. The infection rate slows down significantly and a herd-immunity is achieved after the combined infections reach a peak when the symptomatic infections have crossed ≈ 12% of the total population.

Lock-down had been the most important non-pharmaceutical intervention against COVID-19. However, because of the economic losses caused by the lock-down there have been several proposals to let the infection take its natural course, which would theoretically slowdown sufficient population is infected, popularly known as the attainment of the herd-immunity. The estimates for the population fraction at which the herd-immunity is achieved [24] for European countries for example, had been around 70%. However, specifically for COVID-19, because of the large fraction of asymptomatic infections, estimates for the infection fatality rate, which considers both the asymptomatic and the symptomatic infections, were also made [25]. However, none of these have been made on an integrated framework, and these estimates use the asymptomatic to symptomatic ratio derived from other sources. Our model which is developed on an organic framework which can both estimate the fraction of asymptomatics, and make predictions for the infections, was used for shedding light on herd-immunity. Our results show that the herd-immunity, defined as the fraction of population at which symptomatic infections reach a peak and beyond which begin decreasing could be achieved at 12–25% of the population as illustrated in Fig 11) (Table 2). These estimates for herd-immunity which are in single digit percentages only seem contradictory to estimates of 50-60% [25] until one realises the large fraction of the infections are asymptomatic accounting for a total infection of 50-56% of the population (Table 2). Thus our model allowed us to make estimates both for the hidden-asymptomatics and the herd-immunity, and the fraction of the symptomatics who will burden the health care system.

**Table 2:** The details of the peak of infections extracted using relevant parameters for the COVID-19 dynamics in the different countries and under a hypothetical no-lock-down scenario.

Country	*I*_max_	*M*_max_	Ic (when *I*_max_)	Ic (when *M*_max_)	Mc (when *I*_max_)	Mc (when *M*_max_)
**Italy**	19%	65%	30%	16%	62%	75%
**France**	13%	63%	28%	12%	58%	69%
**Japan**	15%	35%	31%	19%	50%	65%
**Switzerland**	23%	50%	38%	25%	60%	72%

*I*_max_ and *M*_max_ denote peak of the active infection and *A* + *I* curves respectively. *I_c_* denotes cumulative infection which is tabulated for when *I*_max_ and *M*_max_ is achieved in columns 4 and 5 respectively.

## Conclusion

In conclusion, as a part of our analysis, we are able to provide a method for estimating the asymptomatic fraction of the population. Finally, by fitting our model to data from countries where the pandemic appears to have peaked, we are also able to estimate the level of herd-immunity. We are able to show that herd-immunity is achieved at levels of 10% to 25%, far lower than the levels suggested in the literature. We find that the *SAIR* model can be readily adapted to incorporate the effects of lock-down and the solution to the system of equations bears striking resemblance to the real-world data. The formal solution allows one to evaluate the effect of lock-down as a policy tool and can also be integrated into other frameworks which study the economic consequences of the lock-downs.
